# Effects of Periodontal Endoscopy-Assisted Nonsurgical Treatment of Periodontitis: Four-Month Results of a Randomized Controlled Split-Mouth Pilot Study

**DOI:** 10.1155/2022/9511492

**Published:** 2022-01-17

**Authors:** Christian Graetz, Johanna Sentker, Miriam Cyris, Susanne Schorr, Claudia Springer, Karim M. Fawzy El-Sayed

**Affiliations:** ^1^Clinic of Conservative Dentistry and Periodontology, University Hospital of Schleswig-Holstein, Campus Kiel, Kiel, Germany; ^2^Oral Medicine and Periodontology Department, Faculty of Dentistry, Cairo University, Giza, Egypt

## Abstract

**Objective:**

Although the therapeutic effects of nonsurgical periodontal therapy (NSPT) are well established, the clinical benefits of the additional use of periodontal endoscopy (PE) remain controversial. Therefore, this randomized controlled split-mouth pilot study evaluated the effect of NSPT using PE versus NSPT without *n*PE on bleeding on probing (BOP) in sites with probing depth (PD)≥4 mm (primary outcome), PD, clinical attachment level (CAL), number of hard deposits (HDs), and treatment time per tooth (TrT).

**Methods:**

Two calibrated operators performed NSPT in twenty periodontitis patients, randomized into two quadrants for PE or *n*PE treatment. BOP, PD, and CAL were recorded at the first visit for NSPT (*T*0) and during reevaluation (*T*1: mean (SD) 119.7 (24.6) days after *T*0). The average TrT and the number of sites with HDs were documented at *T*0.

**Results:**

For BOP, no significant differences were found at the patient's level (10/10 (male/female); aged 54.3 (10.9) years) neither within or between the groups. At tooth surface level, a lower number of surfaces with BOP (*p*=0.026) was observed in *n*PE. CAL and PD improved significantly during NSPT in both groups (*p* ≤ 0.001), with higher PD reduction (*p* < 0.001) and CAL gain (*p* < 0.001) in *n*PE. There are significantly longer TrT (*p* < 0.001) and more surfaces with subgingival HDs evident in PE at T0 (*p*=0.001).

**Conclusion:**

Whereas subgingival HDs can be visually detected with PE during NSPT, no additional clinical benefits regarding BOP, PD, or CAL were notable compared to conventional systematic periodontal instrumentation. Additionally, PE-assisted NSPT required a longer treatment time.

## 1. Introduction

Advanced periodontitis remains to be one of the primary causes of tooth loss [1]. Nonsurgical periodontal therapy (NSPT) relies primarily on mechanical biofilm and calcified hard deposit (HD) removal [2]. Although its therapeutic effect seems predictable [3], the presence of deep periodontal pockets with limited accessibility [4, 5] may negatively affect the outcome of NSPT, requiring additional flap surgery [6]. It was hypothesized that when flap surgery is contraindicated, periodontal endoscopy (PE) could provide a good sulcus visualization tool, improving the efficacy of nonsurgical subgingival periodontal instrumentation [7, 8].

The primary component of the periodontal endoscopic system is an imaging system with a fiber optic bundle, allowing for visualization of the submarginal area of the periodontium, the root surfaces, and the soft tissue lining [9, 10]. The fiberoptic bundle is connected to a hand instrument (“explorer”), which after being inserted into the sulcus emits a beam of light for root surface illumination and subsequently transmits the images back to a display screen with a magnification up to 48x [11]. The entire fiber optic complex is protected by a sheath to prevent contamination and is linked to a water pump for flushing the periodontal working area during instrumentation [9, 11].

Still, successful removal of subgingival HDs with PE requires both time and training [11, 12]. Recent clinical trials demonstrated a substantial therapeutic effect of PE in sites with a probing depth (PD) > 6 mm compared to conventional NSPT [8, 13]. In contrast, a systematic review and meta-analysis [7] found no significant improvements in bleeding on probing (BOP), gingival inflammation (GI) or PD with PE-assisted NSPT. Given these inconsistent findings, we designed this pilot study as a randomized controlled clinical split-mouth study to assess whether PE-assisted NSPT (test group: PE) has significant advantages over conventional NSPT without PE (control group: *n*PE). Of special interest was whether PE-assisted NSPT (4 ± 1 months) would lead to better clinical outcomes in terms of BOP (primary outcome), PD, clinical attachment level (CAL), and treatment time per tooth (TrT) (secondary outcomes). Furthermore, the study evaluated whether PE-assisted NSPT would remove more HDs than *n*PE and whether a possible difference in outcomes could be accurately detected, employing either a purely visual inspection (with PE) or a limited visual-tactile inspection (in *n*PE).

## 2. Methods

### 2.1. Study Population and Recruitment

All procedures were performed in accordance with the ethical standards of the institutional and/or national research committee (IRB: D509/18) and with the 1964 Helsinki declaration and its later amendments or comparable ethical standards. As the current investigation was designed as a pilot study, no sample size calculation was performed a priori. Still, a post hoc power analysis was performed for the study's primary outcome, namely, BOP. Fifty-two patients scheduled for periodontal therapy at the Department of Periodontology at Kiel University were recruited between September 2019 and March 2020. Inclusion criteria were as follows: (1) age ≥18 and ≤70 years, (2) a recently diagnosed stage III or IV generalized periodontitis according to the 2018 classification [14], (3) *n* ≥ 16 scorable teeth without root caries (diagnosed via radiographs and by clinical visual and tactile methods), (4) availability for NSPT and reevaluation during the next 4 ± 1 months, (5) no physical or mental impairment, (6) not taking medications that influence salivary flow, (7) no special dietary restrictions, and (8) an informed consent to be treated during dental education.

Participants were excluded if they (1) presented with oral diseases other than the defined periodontal disease (e.g., forms of acute necrotizing ulcerating periodontitis or stage I or II periodontitis), (2) had systemic diseases that could influence the therapeutic success (e.g., uncontrolled diabetes mellitus or a tumor in the hard or soft oral tissue) or specific conditions that had to be treated (e.g., prophylaxis of endocarditis), (3) had periodontal treatment including professional tooth cleaning within the previous 12 months, (4) were aware of being pregnant or were breastfeeding, and (5) denied consent to be treated during the study with the integrated use of PE. Patients were included consecutively, as the aim of the study was to compare the effect of PE versus *n*PE during NSPT without influencing the internal procedure.

**Figure 1:**
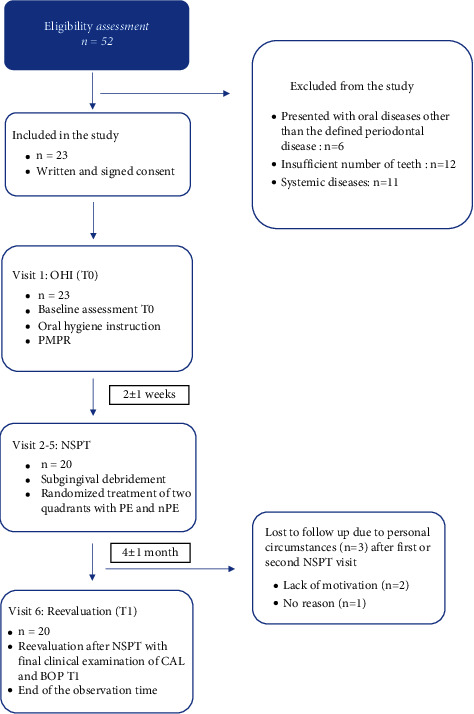
Flowchart of the patients' recruitment and treatment protocol during the study. *T*0: initial treatment visit, *T*1: reevaluation after the treatment phase of nonsurgical periodontitis therapy (NSPT), either quadrant treatment with periodontal endoscopy (PE) or quadrant treatment without periodontal endoscopy (*n*PE) in terms of bleeding on probing (BOP); clinical attachment level (CAL), hygiene instruction (OHI), and supragingival professional mechanical plaque removal (PMPR).

In accordance with the abovementioned inclusion and exclusion criteria, 23 patients were included, of whom three dropped out after the first or second NSPT visit (Figure 1). All participants were informed about the study and gave written informed consent before the start of the investigation. Before initiating the clinical trial, an internal calibration for measuring periodontal parameters was conducted by one dentist (K. F. E.) for all investigators, and test-retest exercises were performed with five nonstudy-related subjects in order to assess interexaminer calibration. A deviation of ±1 mm for PD and CAL was considered acceptable.

Likewise, all data from the evaluations before NSPT (*T*0) and reevaluations of NSPT (*T*1) were conducted by only two calibrated investigators (M. S. and K. F. E.), using a periodontal probe (PCPUNC15, Hu-Friedy, Chicago, IL, USA). Aside from this periodontal probe, the tips of the sonic scaler and/or curettes, and dental loupes at 2.5x magnification at *T*0, no additional devices were utilized to detect HDs in the *n*PE group (PE was used for HD detection in the test group).

### 2.2. Supragingival Professional Mechanical Plaque Removal (PMPR) and Oral Hygiene Instruction (OHI)

All subjects received center-standard instructions regarding individual oral hygiene, with a focus on interdental hygiene (IDH) with interdental brushes (IDBs) and PMPR. Although plaque indices were regularly documented, data were not recorded for every patient at the same time and were not recorded by calibrated examiners; hence, plaque indices were not included in the analysis. Patients were taught how to correctly use a toothbrush (case-by-case decision: a manual toothbrush or oscillating-rotating toothbrush) twice a day and IDBs with fitted sizes for each interdental space to control inflammation while preventing any possible damage to the teeth and soft tissues. The baseline periodontal examination (*T*0) was determined during the first visit for the initial periodontal therapy with PMPR and OHI.

### 2.3. Nonsurgical Periodontal Therapy Phase

According to the internal guidelines of the center-focused dental education, NSPT was implemented at individualized intervals in a quadrant manner over 4 to 6 weeks (mainly depending on scheduling priorities of the center and patients). Two investigators (C. G. and C. S.), with more than 14 years of professional experience in periodontology, used the following instruments: (1) Gracey curettes 5/6, 7/8, 11/12, and 13/14 (American Eagle Instruments, Missoula, MT, USA) with regular and small shapes and (2) an air pressure sonic scaler (Synea, W&H, Bürmoos, Austria) at level 2 (‘medium' amplitude) with a straight slimline tip and round cross section (1AP, W&H, Bürmoos, Austria). The investigators as well as the quadrants to be treated were randomized via https://www.random.org for the type of therapy (subgingival instrumentation with PE (test) or without PE (control)). According to the current European guidelines for the treatment of periodontitis [2], both instrument groups, curettes and power-driven scalers, were equally available and were used according to the practitioner's choice. Consequently, individuals were treated with the same treatment modality (instrumentation with PE or *n*PE) for each pair of diagonally positioned quadrants by the same investigator (in total, 10 patients per investigator). With the help of the tested PE system (Perioscopy^®^, Danville Materials, Zest Dental Solutions, Carlsbad, CA, USA), the operators located HDs or other pathologies visually through subgingival magnified images on the investigated root surface in real time and could remove them.

Both investigators (C. G. and C. S.) participated in the same PE training program [11] and received both general instructions regarding the manufacturer's guidelines and practical sessions in vivo. Both operators were familiar with PE and had used it in routine clinical practice for more than one year. Neither of these operators was blinded to the group assignments or data collected, whereas the investigators at T0 and T1 (M. S. and K. F. E.) were blinded to the quadrants allocated to each treatment (PE or *n*PE). In PE treatment, the clinical endpoint of subgingival instrumentation was defined as the time when no more HDs were detectable visually. In the *n*PE group, both investigators stopped instrumentation individually when a clean (smooth) root surface was perceived using the periodontal probe, tips of the utilized sonic scaler, and/or curettes [15, 16]. All subgingival treatments (*n*PE and PE) were performed under local anesthesia with a professional assistant. In addition to extractions (*n* = 30 teeth, excluded from analysis), no other treatments, with a profoundly direct impact on the treatment outcomes, were performed during the NSPT sessions. No antibiotics were prescribed and no antibacterial agents were used by the dentist during/after NSPT or by the patient at home.

### 2.4. Reevaluation

Reevaluation (*T*1) was performed by two calibrated investigators (M. S. and K. F. E.) for all subjects 4 ± 1 months after *T*0. The reevaluation included, among other aspects, scoring of BOP, PD, and CAL, marking the end of our observation period.

### 2.5. Outcome Variables

The number of teeth sites with PD ≥ 4 mm showing BOP at *T*0 and *T*1 was defined as the primary outcome, while PD, CAL, TrT, and the number of sites with detectable HDs served as secondary outcomes in the two groups (PE or *n*PE). The primary outcome was set according to the general opinion that the presence of pockets ≥4 mm with BOP is the cut-line value when NSPT treatment becomes necessary [2]. A persistently positive BOP score may be a factor for an increased risk for further periodontal tissue destruction [17]. BOP (*T*0 and *T*1) was evaluated at six sites per tooth; hence, interproximal sites were assessed from the buccal both and oral sites. After PD (evaluated at six sites in mm, *T*0 and *T*1) was measured in each quadrant consecutively, using a periodontal probe (PCPUNC15, Hu-Friedy) with 0.2–0.25 N, a positive BOP score was given when bleeding occurred within 10 s. Gingival recession (GR; distance between the cementoenamel junction and the gingival margin) in mm (*T*0 and *T*1) was also evaluated at six sites per tooth using a periodontal probe (PCPUNC15, Hu-Friedy). CAL was calculated as the sum of PD and GR.

TrT for subgingival instrumentation of all teeth from both quadrants of each group (PE or *n*PE) was measured as the sum of the treatment duration, including the time the respective operator needed to change instruments. Afterward, the average TrT per tooth was calculated.

Although the operators were trained in periodontitis treatment and were familiar with utilizing PE [11], timely categorization of the patients was performed to additionally reveal a possible operator learning curve that might have occurred as a result of frequently using PE during the study period. For this purpose, patients were divided into three groups based on the treatment order during the study's observation period. The first seven subjects constituted the early-treatment group, the next seven subjects constituted the middle-treatment group, and the final six subjects constituted the late-treatment group.

Finally, the number of sites with detectable HDs was determined separately for the two methods, with or without PE (T0). HDs were evaluated at six sites per tooth (mesiobuccal, mid-buccal, distobuccal, distooral, midoral, and mesiooral) during the NSPT visits by two calibrated investigators (C. G. and C. S.).

### 2.6. Statistical Analysis

Statistical analysis was performed in a multilevel manner at the tooth surface level and patient level. At the patient level, the mean (SD) of the treatment group (PE or *n*PE) per patient was used for PD and CAL; for the parameter BOP on sites with PD ≥ 4 mm, the average mean value of BOP was used. Data normality was tested by Kolmogorov-Smirnov and Shapiro-Wilk tests. The data for BOP, PD, and CAL at the tooth surface level at T0 and T1 were not normally distributed (Kolmogorov-Smirnov test/Shapiro-Wilk test: (*p* < 0.001)/(*p* < 0.001)). Therefore, changes from *T*0 to *T*1 within groups were assessed using the Wilcoxon nonparametric test, and between-group differences were assessed using the Mann–Whitney *U* or Kruskal-Wallis test. Subanalyses were performed by calculating BOP, PD, and CAL changes for the different types of teeth and for different locations in the oral cavity. Additionally, TrT and the number of sites with HD per group were analyzed.

All statistical tests were two-sided, and a level of *p* < 0.05 was considered to be significant with Bonferroni correction for multiple comparisons. Only subjects with complete data at T0 and T1 were included (*n* = 20), whereas no intention-to-treat analysis was used.

A linear regression analysis at the patient level was performed to assess the associations among predictors (age, smoking, diabetes mellitus, treatment order group, PE/*n*PE group, operator, periodontitis staging, periodontitis grading, number of teeth at *T*0, number of sites with BOP at *T*0, average PD at *T*0, average CAL at *T*0, and number of sites with HD at *T*0) and the mean BOP on sites with PD ≥ 4 mm at *T*1 (dependent variable). Similar was done on tooth surface level with the help of multinomial logistic regression (dependent variable: dichotomy BOP (yes or no) on sites with PD ≥ 4 mm at *T*1). Predictors for the regression analysis were selected by the influence assumed by the investigators and not by previous statistical testing. Regression coefficients, standard errors (SEs), *p* values, and 95% confidence intervals (CIs) were used as effect estimates.

## 3. Results

### 3.1. Sample Characteristics

Twenty patients (males/females: 10/10) with an average age (SD) of 54.3 (10.9) years and a total of 487 teeth (previously lost teeth: *n* = 153) completed the study (PE/*n*PE: 250/237). At *T*0, the majority of the patients showed stage III (*n* = 17) and grade B periodontitis (*n* = 11) (Table 1). No adverse events were reported during the observation period of 119.7 (24.6) days. During NSPT (*T*0-*T*1), 30 teeth were extracted and excluded from the analysis (*n*PE/PE: 20/10). Therefore, the average tooth number per patient was 23.8 (3.8) at *T*0 and 22.5 (5.3) at *T*1. One patient had two dental implants at T0 (both in *n*PE), which were not included in the analysis.

Of the 457 teeth included in the study, 220 were anterior teeth (*n*PE/PE: 109/111), 128 (*n*PE/PE: 65/63) were premolars, and 109 (*n*PE/PE: 56/53) were molars. The majority of all analyzed molars (*n* = 71) showed no furcation involvement (FI) or FI degree 1 at *T*0 (*n*PE/PE: 34/37). Twenty-two molars showed FI degree 2 (*n*PE/PE: 15/7), and 16 molars showed FI degree 3 (*n*PE/PE: 7/9).

Of all tooth surfaces (*n*PE/PE: 1380/1362), 484 sites (PE: 486) in the *n*PE group showed PD ≤ 3 mm at *T*0. For all other sites (*n*PE/PE: 896/876), corresponding to 217 *n*PE-treated and 210 PE-treated teeth, respectively, there were no statistically significant differences between groups at *T*0 regarding BOP, PD, or CAL (Table 2). The median number of sites with PD ≥ 4 mm per patient was (Q25%; Q75%) 44.0 (35.0; 59.5) for the *n*PE group (PE: 48.5 (31.5; 53.8); *p*=0.820).

### 3.2. Clinical Treatment Effects

Inter- and intragroup analyses during different treatment stages (*T*0 and *T*1) were conducted to assess the treatment effects regarding BOP, CAL, and PD. Intraexaminer reproducibility was not assessed. Interexaminer reproducibility was 86% for PD and 79% for CAL but was not assessed for the BOP.

In general, at the tooth surface level, from *T*0 to *T*1, for both the *n*PE and PE groups, BOP, PD, and CAL decreased significantly (Table 2, *p* < 0.001). For CAL and PD, a greater attachment gain and a higher PD reduction were observed in the *n*PE group (CAL: *p*=0.002) and (PD: *p*=0.038; Table 2). The number of sites with BOP at *T*1 after NSPT was significantly lower in both groups. In the *n*PE group (Table 2; *p*=0.026), 25.6% (*n* = 229) of all sites with BOP at *T*0 improved versus 21.8% (191) in the PE group (*p*=0.114). At *T*1, there were significantly fewer tooth surfaces with a positive BOP in the *n*PE group compared to the PE group. Post hoc power analysis of this observed effect was 47%.

When controlling for the treatment effects at the patient level, no significant differences were found for BOP between *T*0 and *T*1, whereas PD reduction and the CAL gain between *T*0 and *T*1 were significantly improved in both treatment groups (Table 2, *p* ≤ 0.05).

At the tooth surface level, a subgroup analysis (Table 3) showed that teeth with a PD of 4–6 mm at *T*0 showed significantly higher CAL gain in the *n*PE group than in the PE group (*p*=0.014). PD reduction and CAL gain in the lower jaw were significantly higher in the *n*PE group than in the PE group (*p* ≤ 0.05, Table 3), with a significantly smaller number of residual pockets with PD ≥ 5 mm detected following *n*PE therapy at *T*2 (number of residual PD in the *n*PE/PE group: 51 (7.9%)/80 (12.8%), *p*=0.004). Among tooth types (anterior, premolar, and molar), no significant differences in BOP, PD, and CAL were detectable, whereas, in all mesial surfaces, the *n*PE group demonstrated a larger treatment effect for all parameters (PD, CAL, BOP, and the number of residual pockets with PD ≥ 5 mm) than the PE group (*p* ≤ 0.05, Table 3).

According to the regression analysis at the patient level, no predictor studied for BOP on sites with PD ≥ 4 mm at T1 was significant (Table 4). At tooth surface level (Table 5), the treatment group (PE; *p*=0.015), the tooth type (anterior tooth *p* < 0.001), the presence of BOP (*p*=0.019), the jaw (maxilla; *p* < 0.001), and the presence of hard deposits (*p*=0.027) at *T*0 were significant predictors. The regression coefficient (*B*) identified that the predictors *n*PE group (*B* = −0.326), anterior teeth (*B* = −0.754), and no BOP at *T*0 (*B* = −0.326) had a positive influence on BOP reduction after NSPT, while the other significant predictors' maxilla (*B* = 0.401) and the presence of HDs (*B* = 0.48) had a negative effect (BOP at *T*1).

### 3.3. Treatment Time

TrT differed significantly between the two treatment groups, with (median (Q25%; Q75%): 2.1 (0.3; 3.2)) min longer TrT per tooth for the PE group than for the *n*PE group (median (Q25%; Q75%) *n*PE/PE: 2.0 (1.5; 3.0)/3.8 (1.9; 5.1) min per tooth; *p* < 0.001). Additionally, a significant intergroup difference (*p* < 0.001) and intragroup difference were obvious among the early- (median (Q25%; Q75%) TrT in *n*PE/PE: 2.0 (1.7; 3.8)/4.6 (4.4; 6.6) min per tooth; *p* < 0.001), middle- (median (Q25%; Q75%) TrT in *n*PE/PE: 1.7 (1.0; 2.9)/3.6 (1.9; 4.8) min per tooth; *p* < 0.001), and late-treatment subgroups (median (Q25%; Q75%) TrT in *n*PE/PE: 1.7 (1.5; 3.0)/1.8 (1.7; 4.7) min per tooth; *p* < 0.001) in both the *n*PE and PE groups. Irrespective of the treatment modality (PE or *n*PE), operators treated all teeth in a significantly shorter time (median (Q25%; Q75%): 2.0 (0.5; 3.7)) min per tooth, in the late-treatment group than in the earlier treatment groups (*p* < 0.001).

### 3.4. Hard Deposit Detection

Among all sites (*n*PE/PE: *n* = 1380/*n* = 1362), HDs were detected in 14% of the sites evaluated with PE (*n*PE: 6.2%). In the PE group, significantly more subgingival HDs were detected than in the *n*PE group at *T*0 (number of detectable HDs in the *n*PE/PE groups: 86/191; *p*=0.001). At the patient level, for a similar number of sites with PD ≥ 4 mm at *T*0 (median (Q25%; Q75%) *n*PE/PE: 44.0 (35.0; 59.5)/48.5 (31.5; 53.8); 0.820), more than double the number of detectable HDs per patient was measurable with PE (Table 2; *p*=0.009).

At the tooth surface level (Table 3), significant differences between the PE and *n*PE groups, regarding the number of sites with detectable HDs, were evident between the jaws (*p* < 0.001) and among the different tooth types (*p* < 0.001) and the tooth surfaces (*p* < 0.0001).

## 4. Discussion

Endoscopes are medical tools with great potential for minimally invasive procedures. In periodontology, subgingival diagnosis using PE was initially proposed several years ago [9, 12]. The present pilot split-mouth RCT investigated the clinical effects of two NSPT procedures, PE and *n*PE, on the periodontal clinical parameters BOP, CAL, and PD, on the TrT, and on the number of HDs detected.

Both treatments improved periodontal outcomes, including BOP, PD and CAL, in accordance with the current evidence on NSPT efficacy [18], whereby among other factors, *n*PE was identified as a significant predictor for the absence of BOP at *T*1 (*B* = −0.326, *p*=0.015). This finding is in contrast to a recently published clinical investigation by Naicker, Ngo [8], who observed a reduction in BOP of over 70% in both treatment groups and a significantly lower BOP for PE-assisted NSPT on longer-term follow-ups (at 6, 9, and 12 months). Although in the current split-mouth design risk factors, such as smoking, were controlled (45% of all included patients were smokers, and former smokers were classified as nonsmokers when they had not smoked for at least five years [19]), diabetes or self-performed oral hygiene may have influenced our treatment results [20].

Similar to BOP, no significant clinical benefits were detectable at the patient level for PE-assisted NSPT in terms of PD and CAL, with PE requiring a significantly longer TrT. Although, with a TrT of 4.2 min per tooth, the findings were comparable to our recent in vitro results [11], they still remained in contrast to a previously published clinical investigation [21] that demonstrated a significantly longer mean TrT of 19 min per tooth with PE versus 13 min without PE. The longer TrT reported in the latter study could be based on the fact that all teeth evaluated had been previously deemed untreatable due to prosthetic or periodontal reasons, with higher levels of gingival inflammation, mobility, granulation tissues, and carious lesions, which could have affected the operator's instrumentation ability [22]. In the current study, we focused on interventions of the second periodontal therapeutic level [2] and identified a ratio of 1 : 2 for the TrT between groups. The additional time of almost two minutes per tooth seems acceptable, especially during reevaluations for additional treatment of the remaining inflamed sites or during SPT, as mentioned by Osborn et al. [23]. Routine utilization of PE further positively affected TrT. Michaud et al. [21] and Geisinger et al. [12] subanalyzed TrT in relation to patient enrollment (first, middle and last), presuming that operators were more experienced at the end of the study in using PE and were consequently faster. For clinicians who were trained in using the device, the TrT required to achieve subgingival instrumentation decreased over time and approached the duration of hand instrumentation [12, 21]. The current results support these findings, highlighting the fact that the extra TrT required by PE during subgingival instrumentation in the hands of trained operators seems to be negligible [23]. The learning curve in our study indicates that, especially for TrT the continuous use is required and that the operator can effectively accelerate the process when she/he has gained sufficient knowledge, dexterity, and confidence about this technology.

Aside from the system's evident limitations, it remains pivotal to emphasize the fact that a reduction in subgingival microorganisms remains to be the key element for a successful periodontal therapy rather than the absolute removal of HDs [24, 25]. Although complete HDs removal from root surfaces positively affects subsequent healing [26], it does not seem to be absolutely necessary for the correction of microbial dysbiosis, and a comparable tendency for periodontal healing on nearly polished residual HDs was described [27, 28]. The fact remains that the quantitative and qualitative proportions of oral biofilms and HDs that correlate with healthy periodontal conditions are currently unclear [29]. A qualitative categorization (endoscopic calculus index) of the visually detectable HDs, as described by Checchi et al. [30], was not used in our study, and only a quantitative assessment was performed. Another factor possibly limiting the expression of the full range of abilities of PE could rely on the fact that although NSPT was performed mainly on stage III periodontitis patients, it was not guaranteed that more than two sites with a PD greater than 6 mm would be present in each patient. To overcome this limitation, we performed a multilevel analysis of our split-mouth data.

Naicker et al. [8] observed that PE-assisted NSPT resulted in a slight, statistically significant benefit in pockets >6 mm (up to 9 mm) and more radiographic bone gain was noticed around multirooted teeth. We failed to show such clinical benefit, yet, we detected that molars, which are the least accessible, showed the lowest amount of detectable HDs in both groups at T0 (Table 3, *p* < 0.01), in contrast to results by Michaud et al. [21] and Checchi et al. [30]. Experienced operators, including both investigators in the current study, might have acknowledged these difficulties in effectively treating these surfaces while constantly maintaining contact between the tip of the instrument and the tooth [31]. It appears further plausible to conclude that the additional benefits provided by the advanced visualization of subgingival HDs by PE could have been minimal for an experienced operator professionally trained in performing rigorous systematic subgingival periodontal instrumentation, even in the absence of direct visualization of HDs, as in the current study. In this context, a restriction of our study lies in the fact that no tooth extractions were deemed necessary or were undertaken at *T*1 to possibly check the effectiveness of HD removal and relate it to the measured clinical outcomes.

Importantly, the current results cannot be generalized for the following reasons: (1) only patients with stages III or IV periodontitis were included, (2) comprehensive treatment was administered in a university setting, (3) the observation period was short (4 ± 1 months), and (4) treatment was assigned by quadrant. It could be assumed that a longer observation time after the successful removal of HDs would lead to greater clinical attachment gain or at least fewer instances of recurrence requiring surgical periodontal therapy [8]. (5) We investigated the removal of recognizable subgingival HDs without pre- and postobservational quantification. (6) In addition, we did not control for factors such as patient compliance and self-performed oral hygiene. Poor plaque scores or small improvements could have affected our treatment outcomes [2]; however, due to the chosen randomization and split-mouth design, these effects should have been minimized.

## 5. Conclusion

Within the present study's limitations, no clinical benefits were observed at the patient's level for the additional use of periodontal endoscopy in NSPT within four months of observation. Whether the additional benefit of better visualization of hard deposits during subgingival instrumentation provided by periodontal endoscopy is useful in specific therapeutic settings, e.g., during supportive periodontal therapy and in special clinical conditions, including very deep pockets and anatomically difficult conditions, needs to be further studied.

## Figures and Tables

**Table 1:** Demographic and clinical data of the patient cohort.

Baseline characteristics (T0)	
*Sex (n (%))*	
Male	10 (50.0)
Female	10 (50.0)

*Smoking (n (%))*	
Yes	9 (45.0)
No	11 (55.0)

*Diabetes mellitus (n (%))*	
Yes	1 (5.0)
No	19 (95.0)

*Age (years)*	
Mean (SD)	54.3 (10.9)

*Periodontitis stage (n (%))*	
Stage III	17 (85.0)
Stage IV	3 (15.0)

*Periodontitis grade (n (%))*	
Grade *A*	2 (10.0)
Grade *B*	11 (55.0)
Grade *C*	7 (35.0)

*Teeth per patient (n)*	
Teeth per patient (SD) at *T*0	23.8 (3.8)
Teeth per patient (SD) at *T*1	22.5 (5.3)

*Teeth removed during T0-T1 per patient (n)*	
Teeth (SD)	1.3 (2.9)

*Examined teeth (n (%))*	
Present at *T*0	487 (76.1)
Not available at *T*0	153 (23.9)

*Teeth status (n (%))*	
Survived from T0 to T1	457
Removed during T0 to T1	30

*Time from T0 to T1 (days)*	
Mean (SD)	119.7 (24.6)

Standard deviation (SD), frequency (*n*), percentage (%), baseline (*T*0); reevaluation visit after the end of nonsurgical periodontal therapy (*T*1)

**Table 2:** Treatment results, part I (only sites with PD ≥ 4 mm at *T*0).

Variables	Control *n*PE *n* = 896	Test PE *n* = 876	*P* value between the control and the test^*∗*^
Results at the tooth surface level			
*N* of sites with BOP (%) at *T*0	262 (29.2)	254 (29.0)	0.909
*N* of sites with BOP (%) at *T*1	125 (14.0)	156 (17.8)	**0.026**
*N* of sites with improved BOP (%) between *T*0 to *T*1 with BOP at *T*0	229 (25.6)	191 (21.8)	0.114
*P* value *for BOP at T0 vs. T*1^†^	**<0.001**	**<0.001**	
Median (Q25%; Q75%) PD at *T*0	5.0 (4.0; 6.0)	5.0 (4.0; 6.0)	0.250
Median (Q25%; Q75%) PD at *T*1	3.0 (3.0; 4.0)	3.0 (3.0; 4.0)	0.439
Median (Q25%; Q75%) PD change *T*0-*T*1	−2.0 (−2.0; −1.0)	−1.0 (−2.0; −1.0)	**0.038**
*P* value *for PD at T0 vs. T*1^†^	**<0.001**	**<0.001**	
Median (Q25%; Q75%) CAL at *T*0	5.0 (4.0; 7.0)	5.0 (4.0; 7.00)	0.588
Median (Q25%; Q75%) CAL at *T*1	5.0 (3.0; 6.0)	5.0 (4.0; 7.0)	0.059
Median (Q25%; Q75%) CAL change *T*0-*T*1	−1.0 (−2.0; 0)	−1.0 (−2.0; 0)	**0.002**
*P* value for CAL at *T*0 vs. *T*1^†^	**<0.001**	**<0.001**	

Results at the patient level			
Median (Q25%; Q75%) of the *N* of sites with PD ≥ 4 mm (%)/*N* of all sites at *T*0 per patient	44.0 (35.0; 59.5)	48.5 (31.5; 53.8)	0.820
Median (Q25%; Q75%) of the *N* of sites with HD (%)/*N* of all sites at *T*0 per patient	3.5 (2.0; 5.0)	7.5 (3.5; 9.8)	**0.009**
Median (Q25%; Q75%) of the *N* of sites with BOP (%)/*N* of all sites at *T*0 per patient	0 (0; 0.7)	0 (0; 0.7)	0.968
Median (Q25%; Q75%) of the *N* of sites with BOP (%)/N of all sites at *T*1 per patient	0.1 (0; 0.3)	0.1 (0; 0.4)	0.659
Median (Q25%; Q75%) of the *N* of sites with BOP (%)/*N* of all sites change between *T*0 to *T*1 with BOP at *T*0 per patient	0 (−0.5; 0.2)	0.1 (0; 0.4)	0.968
*P* value for BOP at T0 vs. *T*1^†^	0.394	0.495	
Median (Q25%; Q75%) PD at *T*0 per patient	5.0 (4.9; 5.6)	5.1 (4.8; 5.4)	1.000
Median (Q25%; Q75%) PD at *T*1 per patient	3.4 (3.2; 3.9)	3.6 (3.1; 3.8)	0.799
Median (Q25%; Q75%) PD changing *T*0-*T*1 per patient	−2.1 (−1.7; −1.3)	−1.7 (−2.0; −1.3)	0.678
*P* value for PD at T0 vs. *T*1^†^	**<0.001**	**<0.001**	
Median (Q25%; Q75%) CAL at *T*0 per patient	5.5 (5.0; 6.2)	5.4 (5.1; 6.1)	1.000
Median (Q25%; Q75%) CAL at *T*1 per patient	5.3 (4.0; 5.9)	5.0 (4.3; 6.2)	0.659
Median (Q25%; Q75%) CAL changing *T*0-*T*1 per patient	−0.6 (−1.3; −0.3)	-0.5 (−1.2; −0.1)	0.495
*P* value for CAL at *T*0 vs. *T*1^†^	**0.030**	**0.044**	

^*∗*^ Mann–Whitney test; †Wilcoxon test (significant differences in bold). Descriptive statistics and results for comparison between both treatment groups and visits. Median and lower/upper quartiles (Q25%; Q75%); frequency (*n*); percentage (%); clinical attachment level (CAL in mm); hard deposit (HD); nonsurgical periodontal therapy (NSPT); test teeth without periodontal endoscopy treatment (*n*PE); test teeth with periodontal endoscopy treatment (PE); pocket probing depth (PD in mm); baseline (*T*0); reevaluation visit after the end of NSPT (*T*1); bleeding on probing (BOP, all sites) of all surviving teeth (control: *n* = 230; test: *n* = 227).

**Table 3:** Treatment results and part II subgroup analysis at the tooth surface level.

			*N* of sites (%) with HD detected at T0	Median (Q25%; Q75%) PD change T0−T1	Median (Q25%; Q75%) CAL change T0−T1	N of sites (%) with residual PD at T1	N of sites (%) with BOP at T1 (%)
Categorized initial PD	*n*PE	PD ≤ 3 mm	7 (1.4)	0 (0, 0)	1.0 (0, 1.0)	1 (0.2)	38 (7.8)
		PD = 4–6 mm	67 (8.6)	−1.0 (−2.0, −1.0)	**−1.0 (−2.0, 0)** ^*∗*^	77 (9.8)	107 (13.7)
		PD ≥ 7 mm	12 (10.6)	−4.0 (−4.0, −3.0)	−2.0 (−3.0, −1.0)	34 (30.1)	18 (15.9)
	PE	PD ≤ 3 mm	**39 (8.0)** ^‡^	0 (0, 0)	0 (0, 1.0)	6 (1.2)	34 (7.0)
		PD = 4–6 mm	**132 (16.8)** ^‡^	−1.0 (−2.0, −1.0)	−0.5 (−1.0, 1.0)	96 (12.2)	132 (16.8)
		PD ≥ 7 mm	**20 (22.2)** ^*∗*^	−3.0 (−4.0, −2.0)	−1.0 (−3.0, 0)	35 (38.9)	24 (26.7)

Jaw	*n*PE	Upper jaw	40 (6.2)	−1.0 (−2.0, 0)	−1.0 (−1.0, 0)	**51 (7.9)** ^‡^	88 (13.6)
		Lower jaw	46 (6.2)	**−1.0 (−2.0, 0)** ^*∗*^	**−1.0 (−1.0, 0)** ^‡^	61 (8.3)	75 (10.2)
	PE	Upper jaw	**99 (15.9)** ^‡^	−1.0 (−2.0, 0)	0 (−1.0, 1.0)	80 (12.8)	98 (15.7)
		Lower jaw	**92 (12.5)** ^‡^	−1.0 (−2.0, 0)	0 (−1.0, 1.0)	57 (7.7)	92 (12.5)

Tooth type	*n*PE	Anterior teeth	30 (4.6)	−1.0 (−2.0, 0)	0 (−1.0, 1.0)	27 (4.1)	62 (9.4)
		Premolars	25 (6.4)	−1.0 (−2.0, 0)	0 (−1.0, 1.0)	22 (5.6)	46 (11.8)
		molars	31 (9.2)	−1.0 (−2.0, 0)	−1.0 (−1.7, 0)	63 (18.8)	55 (16.4)
	PE	Anterior teeth	**76 (11.4)** ^‡^	−1.0 (−2.0, 0)	0 (−1.0, 1.0)	29 (4.4)	69 (10.4)
		Premolars	**45 (11.9)** ^‡^	−1.0 (−2.0, 0)	0 (−1.0, 1.0)	33 (8.7)	54 (14.3)
		molars	**70 (22.0)** ^‡^	−1.0 (−2.0, 0)	0 (−1.0, 1.0)	75 (23.6)	67 (21.1)

Tooth surface	*n*PE	Mesial	33 (7.2)	**−1.0 (−2.0, 0)** ^*∗*^	**−1.0 (−2.0, 0)** ^*∗*^	**42 (9.1)** ^*∗*^	**60 (13.0)** ^*∗*^
		Middle	17 (3.7)	0 (−1.0, 0)	0 (−1.0, 1.0)	19 (4.1)	36 (7.8)
		Distal	36 (7.8)	−1.0 (−2.0, 0)	0 (−1.0, 0)	51 (11.0)	67 (14.5)
	PE	Mesial	**66 (14.5)** ^‡^	−1.0 (−2.0, 0)	0 (−1.0, 1.0)	63 (13.9)	82 (18.1)
		Middle	**59 (13.0)** ^‡^	−1.0 (−1.0, 0)	0 (−1.0, 1.0)	24 (5.3)	45 (9.9)
		Distal	**66 (14.5)** ^‡^	−1.0 (−2.0, 0)	0 (−1.0, 1.0)	50 (11.0)	63 (13.9)

Treatment order groups	*n*PE	Early-treatment group	23 (5.0)	−1.0 (−2.0, 0)	0 (−1.0, 1.0)	65 (14.1)	73 (15.8)
		Middle-treatment group	36 (6.8)	−1.0 (−2.0, 0)	−1.0 (−2.0, 0)	21 (4.0)	57 (10.8)
		Late-treatment group	27 (6.8)	−1.0 (−2.0, 0)	0 (−1.0, 1.0)	26 (6.6)	33 (8.3)
	PE	Early-treatment group	**73 (15.8)** ^‡^	−1.0 (−2.0, 0)	0 (−1.0, 1.0)	70 (15.2)	73 (15.8)
		Middle-treatment group	**50 (10.4)** ^‡^	−1.0 (−2.0, 0)	−1.0 (−2.0, 0)	26 (5.4)	68 (14.2)
		Late-treatment group	**68 (16.2)** ^‡^	−1.0 (−1.0, 0)	0 (−1.0, 1.0)	41 (9.8)	49 (11.7)

Mann–Whitney test between PE versus *n*PE groups (significant differences in bold): ^*∗*^*p* ≤ 0.05; ^†^*p* ≤ 0.01; ^‡^*p* ≤ 0.001 (Bonferroni adjustment). Descriptive statistics and results for comparison between both groups of treatment for *N* of sites with HD (% of all teeth), median (Q25%, 75%) change in PD between *T*0-*T*1 (in mm), median (Q25%, 75%) change in CAL between *T*0-*T*1 (in mm), *N* of sites with residual pockets at *T*1 (PD ≥ 5 mm), and N of sites with BOP at *T*1 (% all tooth surfaces) of all survived teeth separated by groups of PD (categorized in ≤3 mm, 4–6 mm and >7 mm) at *T*0, jaw location (upper *vs*. lower jaw) and tooth type (anterior teeth, premolars, and molars), tooth surface (mesial, middle, and distal), and treatment order (early, middle, and late). All significant differences with better results in PE/*n*PE are highlighted in bold. Standard deviation (SD); frequency (*n*); percentage (%); clinical attachment level (CAL); hard deposit (HD); nonsurgical periodontal therapy (NSPT); lower/upper quartile (Q25%, Q75%); test teeth without periodontal endoscopy treatment (*n*PE); test teeth with periodontal endoscopy treatment (PE); pocket probing depth (PD); baseline (*T*0); reevaluation visit after the end of NSPT (*T*1).

**Table 4:** Results of linear regression analysis at the patient level.

Variables		*B*	SE	95% CI		*P* value
				Lower limit	Upper limit	
Constant		56,71	13,489			**<0.001**
Age		0,03	0,063	0,911	1,165	0,635
Smoking	No	1,972	2,262	0,085	605,332	0,383
	Yes (reference)	0				

Diabetes mellitus	No	−43,48	0	1,31*E* − 19	1,31*E* − 19	1
	Yes (reference)	0				

Treatment group	*n*PE	−0,589	1,124	0,061	5,024	0,6
	PE (reference)	0				

Operator	One	60,639	6342,559	0	1	0,992
	Two (reference)	0				

Treatment order group	Early-treatment group	48,428	51,672	1,12*E* − 23	1,04*E* + 65	0,349
	Middle-treatment group	33,22	31,023	1,05*E* − 12	6,82*E* + 40	0,284
	Late-treatment group (reference)	0				

Periodontitis staging	Stage III	−40,499	2983,526	0	1	0,989
	Stage IV (reference)	0				

Periodontitis grading	Grade *A*	44,014	2983,527	0	1	0,988
	Grade *B*	45,61	2983,53	0	1	0,988
	Grade *C* (reference)	0				

N of teeth at *T*0		−1,116	0,588	0,103	1,038	0,058
N of sites with BOP at *T*0		−4,733	3,632	7,13*E* − 06	10,862	0,193
Average PD at *T*0		−9,144	5,785	1,27*E* − 09	8,966	0,114
Average CAL at *T*0		10,755	6,016	0,355	6,192	0,074
N of sites with HDs at *T*0		−0,108	0,136	0,688	1,171	0,426

Bleeding on probing (mean BOP) at sites with PD ≥ 4 mm at *T*1 was considered as a dependent variable. Clinical attachment level (CAL); hard deposit (HD); test teeth without periodontal endoscopy treatment (*n*PE); test teeth with periodontal endoscopy treatment (PE); pocket probing depth (PD); baseline (*T*0); reevaluation visit after the end of nonsurgical periodontal therapy (*T*1). Regression coefficient (*B*), standard error (SE), odds ratio (OR): significant at *P* ≤ 0.05 (in bold).

**Table 5:** Results of multinomial logistic regression analysis at tooth surface level.

Variables		*B*	SE	95% CI		*P* value
				Lower limit	Upper limit	
Constant		−1.797	0.455			**<0.001**
Treatment group	*n*PE	−0.326	0.134	0.555	0.938	**0.015**
	PE (reference)	0				

Treatment order group	Early-treatment group	0.188	0.166	0.871	1.673	0.257
	Middle-treatment group	−0.241	0.183	0.549	1.125	0.188
	Late-treatment group (reference)	0				

Jaw	Maxilla	0.491	0.135	1.255	2.128	**<0.001**
	Mandible (reference)	0				

Tooth type	Anterior tooth	−0.754	0.164	0.341	0.648	**<0.001**
	Premolar	−0.314	0.165	0.528	1.01	0.057
	molar (reference)	0				

Tooth surface	Mesial	0.108	0.149	0.833	1.49	0.468
	Middle	−0.007	0.183	0.693	1.421	0.967
	Distal	0				

PD at *T*0		0.085	0.092	0.909	1.303	0.356
CAL at *T*0		−0.042	0.072	0.833	1.103	0.557
Presence of BOP at *T*0	No	−0.362	0.155	0.514	0.942	**0.019**
	Yes (reference)	0				

Presence of HDs at *T*0	No	0.48	0.217	1.055	2.474	**0.027**
	Yes (reference)	0				

Bleeding on probing (dichotomy BOP: yes or no) at sites with PD ≥ 4 mm at *T*1 was considered as a dependent variable (number of included cases: *n* = 1772). Clinical attachment level (CAL); hard deposit (HD); test teeth without periodontal endoscopy treatment (*n*PE); test teeth with periodontal endoscopy treatment (PE); pocket probing depth (PD); baseline (*T*0); reevaluation visit after the end of nonsurgical periodontal therapy (*T*1). Regression coefficient (*B*). Standard error (SE). Odds ratio (OR): significant at *P* ≤ 0.05 (in bold).

## Data Availability

The clinical data used to support the findings of this study are restricted by the Ethics Board Kiel (IRB) in order to protect patient's privacy. Data are available from the corresponding author for researchers who meet the criteria for access to confidential data.
